# High TMEM161A expression drives malignant phenotypes and predicts poor prognosis in colorectal cancer

**DOI:** 10.1016/j.isci.2026.116550

**Published:** 2026-06-27

**Authors:** Kengo Haruna, Norikatsu Miyoshi, Shiki Fujino, Rie Mizumoto, Yuki Toyoda, Rie Hayashi, Mitsunobu Takeda, Yuki Sekido, Tsuyoshi Hata, Atsushi Hamabe, Takayuki Ogino, Mamoru Uemura, Hirofumi Yamamoto, Hidetoshi Eguchi, Yuichiro Doki

**Affiliations:** 1 Department of Gastroenterological Surgery, Graduate School of Medicine, The University of Osaka, Osaka 565-0871, Japan; 2 Department of Innovative Oncology Research and Regenerative Medicine, Osaka International Cancer Institute, Osaka 545-0871, Japan

**Keywords:** TMEM161A, colorectal cancer, prognostic biomarker, cell cycle, cancer stemness

## Abstract

Transmembrane protein 161 A (TMEM161A), also known as adaptive response to oxidative stress-29, has been implicated in cellular stress responses, but its role in colorectal cancer (CRC) remains unclear. Here, we investigated the clinical relevance and functional significance of TMEM161A in CRC, using integrated clinical, experimental, and transcriptomic analyses. Immunohistochemical analysis of 133 surgically resected CRC specimens showed that high TMEM161A expression was associated with worse survival outcomes in our institutional cohort. Functional assays using siRNA-mediated *TMEM161A* knockdown demonstrated marked suppression of malignant phenotypes, including proliferation, migration, invasion, and a stemness-related phenotype. Transcriptomic profiling coupled with gene set enrichment analysis revealed that *TMEM161A* silencing downregulated cell cycle- and growth-related pathways while activating stress response and checkpoint signaling programs. Collectively, these findings support a role for TMEM161A in malignant behavior in CRC and suggest that TMEM161A may serve as a prognostic biomarker candidate and a potential therapeutic target, linking tumor growth with cellular stress responses.

## Highlights

• High TMEM161A expression predicts poor prognosis in colorectal cancer
• TMEM161A silencing suppresses growth and invasion-related phenotypes
• RNA-seq/GSEA showed E2F and G2/M down, p53 up after TMEM161A silencing
• TMEM161A is a candidate prognostic biomarker and therapeutic target in CRC

## Graphical abstract

**Figure:**
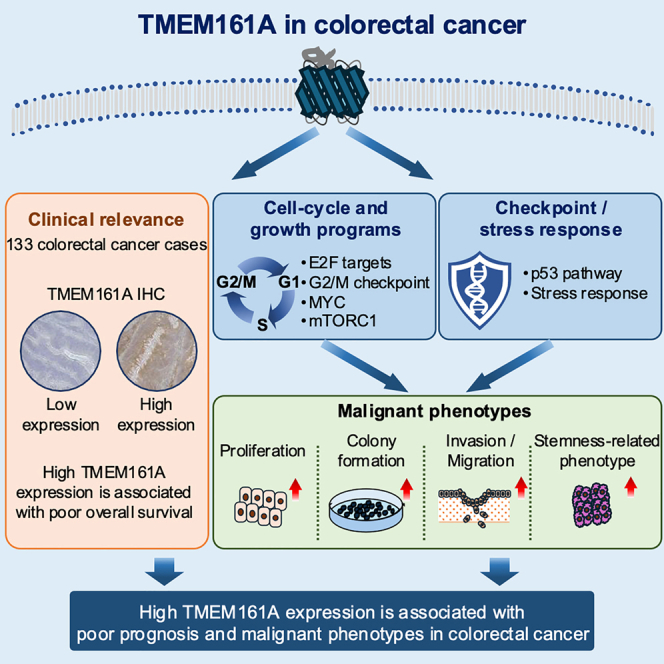

## Teaser

Health sciences; Biological sciences; Cancer systems biology; Transcriptomics

## Introduction

Cancer remains one of the leading causes of death worldwide, and its incidence and mortality are predicted to continue to increase. In 2022, approximately 20 million new cancer diagnoses and 9.7 million cancer-related deaths were reported worldwide, and current Global Cancer Observatory projections suggest that approximately 20% of individuals will develop cancer during their lifetime, with approximately 11% of men and 8% of women predicted to die from the disease. The global cancer burden is projected to increase further, reaching approximately 35 million new cases by 2050.^1^ Among all malignancies, colorectal cancer (CRC) ranks third in global incidence and second in cancer-related mortality, and it accounted for approximately 1.9 million new diagnoses and 930,000 deaths worldwide in 2022.^1^ The etiology of CRC is complex and multifactorial, involving genetic predispositions, such as Lynch syndrome and familial adenomatous polyposis, and a wide array of environmental factors, including diet, obesity, physical inactivity, microbiome alterations, and early life exposure.^2^ CRC is a molecularly heterogeneous disease that develops through multiple routes of genomic instability, including chromosomal instability, mismatch repair deficiency with microsatellite instability (MSI), and a smaller subset of hypermutated tumors with polymerase proofreading defects^3^^,^^4^ Recent large-scale genomic and integrated transcriptomic analyses have further refined this landscape, identifying recurrent driver alterations involving *APC*, *KRAS*, *TP53*, and *BRAF* and showing that these molecular contexts are associated with biologically and clinically distinct subgroups.^3^^,^^4^ In addition, transcriptome-based classification frameworks have advanced beyond conventional clinicopathological grouping, indicating that CRC comprises molecular subclasses with differing prognostic and biological features.^4^ In this context, the continued identification and characterization of CRC-associated genes remain essential for improving molecular stratification and developing new biomarkers and targeted therapies.

We previously conducted an RNA sequencing (RNA-seq)-based transcriptomic analysis, comparing normal colorectal epithelial cells, CRC cell lines, and primary cultured CRC cells.^5^ From this analysis, we extracted genes that met the following criteria: (1) significantly higher expression in CRC than in normal epithelial cells, and (2) a differential expression *p* value of <0.005. Among the upregulated genes, we focused on transmembrane protein 161 A (*TMEM161A*), which has been previously implicated in other cancer types but not in CRC.

*TMEM161A*, also known as adaptive response to oxidative stress-29 (*AROS-29*), was first reported in 2006 as a gene upregulated in cells adapted to oxidative stress. Its overexpression reduces DNA damage and apoptosis, suggesting a role in protecting against oxidative stress-induced damage.^6^ Notably, this finding was based on experiments using the human osteosarcoma cell line Saos-2. TMEM161A is overexpressed in non-small cell lung cancer compared with normal lung tissue.^7^ However, to date, no study has investigated its role in CRC. Therefore, in this study, we aimed to investigate the biological function of TMEM161A in CRC.

## Results

### High TMEM161A expression correlated with poor prognosis in patients with CRC in our institutional cohort

**Figure 1:**
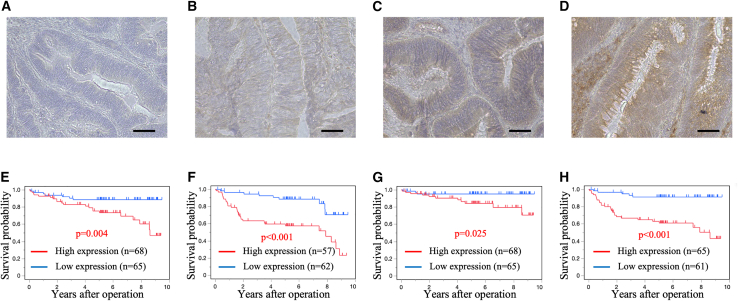
TMEM161A immunohistochemistry and survival in colorectal cancer (A–D) Representative hot-spot fields at 200× magnification (DAB-hematoxylin). Scale bars are 100 μm. Staining intensity was scored by cytoplasmic signal: (A), 0; (B), 1+; (C), 2+; (D), 3+. Cases were dichotomized as low (0–1) and high (2–3) for subsequent analyses. (E–H) Kaplan-Meier curves comparing the high- and low-expression groups for the prespecified survival endpoints: (E) overall survival (high, *n* = 68; low, *n* = 65), (F) disease-free survival (high, *n* = 57; low, *n* = 62), (G) cancer-specific survival (high, *n* = 68; low, *n* = 65), and (H) recurrence-free survival (high, *n* = 65; low, *n* = 61). Survival curves were compared using the log rank test. Group sizes and *p* values are shown in each plot.

To assess the clinical relevance of TMEM161A expression in CRC, immunohistochemical (IHC) analysis was performed using tumor specimens from 133 patients who underwent curative resection at our institution. TMEM161A expression was semi-quantitatively evaluated based on the cytoplasmic staining intensity in tumor hotspots and classified into four categories (scores 0–3). Cases were subsequently dichotomized into low expression (scores 0–1, *n* = 65) and high expression (scores 2–3, *n* = 68) primarily based on pathological interpretability, separating absent/weak staining from moderate/strong staining (Figures 1A–1D).

Comparison of the clinicopathological variables revealed no significant differences between the TMEM161A-high and TMEM161A-low expression groups (Table 1). However, Kaplan-Meier survival analyses demonstrated that high TMEM161A expression was significantly associated with worse overall survival (OS) (*p* = 0.004), disease-free survival (*p* < 0.001), cancer-specific survival (*p* = 0.025), and recurrence-free survival (*p* < 0.001) (Figures 1E–1H).

**Table 1:** Comparison of clinicopathological characteristics between high- and low-TMEM161A expression groups among patients with colorectal cancer

Factors	Low expression (*n* = 65)	High expression (*n* = 68)	*p* value
Age (years, median [range])	67 [40–85]	69 [41–86]	0.586
Sex (male/female)	31/34	25/43	0.223
Tumor location (right/left)	11/54	18/50	0.212
Histology (tub1, tub2/por, sig, muc)	55/10	55/13	0.650
pT (Tis, 1, 2/3, 4)	28/37	27/41	0.727
pN (0/1, 2, 3)	42/23	42/26	0.858
pM (0/1)	61/4	65/3	0.714
Venous invasion (−/+)	37/28	34/34	0.488
Lymphatic invasion (−/+)	33/32	32/36	0.730
pStage (0, I, II/III, IV)	42/23	43/25	1.000

tub1/2, well/moderately differentiated tubular; por, poorly differentiated; sig, signet-ring; muc, mucinous.

In univariate Cox regression analysis, high TMEM161A expression was associated with an increased hazard ratio (HR) (HR = 3.251; 95% confidence interval [CI], 1.380–7.654; *p* = 0.007). Multivariate analysis confirmed that high TMEM161A expression remained an independent predictor of poor OS (HR = 3.461; 95% CI, 1.435–8.356; *p* = 0.006), along with male sex (*p* = 0.003), elevated serum CA19-9 levels (*p* = 0.005), and distant metastasis (*p* = 0.017) (Table 2).

**Table 2:** Univariate and multivariate Cox regression analyses for overall survival of patients with colorectal cancer

Variable	Univariate analysis			Multivariate analysis		
	HR	95% CI	*p* value	HR	95% CI	*p* value
Age (≥70/<70)	1.733	0.822–3.652	0.148	–	–	–
Sex (male/female)	2.642	1.123–6.237	0.026	4.173	1.638–10.633	0.003
CEA (≥5/<5 ng/mL)	2.058	0.966–4.383	0.062	–	–	–
CA19-9 (≥37/<37 U/mL)	5.228	2.377–11.502	<0.001	3.731	1.474–9.441	0.005
Tumor location (right/left)	0.554	0.192–1.599	0.275	–	–	–
Histological type (por, sig, muc/tub1, tub2)	1.065	0.404–2.804	0.899	–	–	–
pT (3, 4/Tis, 1, 2)	3.177	1.286–7.849	0.012	1.795	0.642–5.021	0.265
pN (1, 2, 3/0)	3.312	1.528–7.178	0.002	2.130	0.826–5.494	0.118
pM (1/0)	12.518	4.410–35.530	<0.001	4.973	1.331–18.584	0.017
TMEM161A expression (high/low)	3.251	1.380–7.654	0.007	3.461	1.435–8.356	0.006

HR, hazard ratio; CI, confidence interval; CEA, carcinoembryonic antigen; CA19-9, carbohydrate antigen 19–9; tub1/2, well/moderately differentiated tubular; por, poorly differentiated; sig, signet-ring; muc, mucinous.

### *TMEM161A* expression is efficiently silenced by siRNA in CRC cell lines

To investigate the functional role of TMEM161A in CRC, we selected two CRC cell lines, HT29 and LIM1215, which exhibit relatively high endogenous TMEM161A expression. These cells were transfected with *TMEM161A*-targeting siRNA or negative-control siRNA. The knockdown efficiency was evaluated by quantitative reverse-transcription PCR (RT-qPCR) and western blotting analysis.

**Figure 2:**
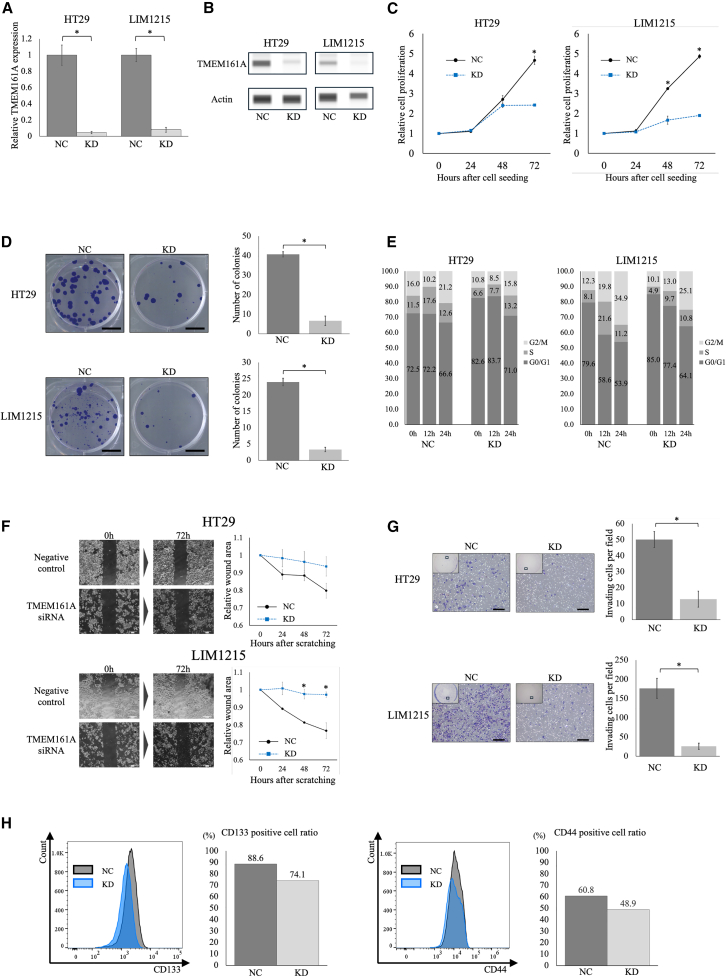
Functional effects of TMEM161A silencing in colorectal cancer cells (A) RT-qPCR analysis of TMEM161A mRNA in HT29 and LIM1215 cells at 48 h after transfection with negative-control siRNA (NC) or *TMEM161A* siRNA (KD). Expression was normalized to GAPDH and is shown relative to NC. Bars represent the mean ± SD of three technical replicates from RNA obtained from a single well for each condition. ∗*p* < 0.05, two-tailed unpaired Student’s *t* test. (B) Representative Simple Western image showing reduction of TMEM161A protein levels in HT29 and LIM1215 cells at 48 h after transfection with NC or KD. Actin is shown as a loading control. (C) Cell growth curves (CCK-8) measured at 0, 1, 2, and 3 days after seeding in HT29 and LIM1215 cells transfected with NC or KD. Proliferation is shown as the fold change in absorbance relative to the 0-h time point. Data are presented as the mean ± SD of five replicate wells per condition. ∗*p* < 0.05, two-tailed unpaired Student’s *t* test comparing NC and KD at the corresponding time point. (D) Colony formation assay in HT29 and LIM1215 cells transfected with NC or KD. Cells were seeded at 100 cells/well and cultured for 10 days. Colonies containing ≥50 cells were fixed with methanol, stained with 0.5% crystal violet, and counted under a stereomicroscope. Representative well images and colony counts are shown (scale bars, 1 cm). Data are presented as the mean ± SD of colony counts from three parallel wells per condition in a representative experiment. ∗*p* < 0.05, two-tailed unpaired Student’s *t* test. (E) Cell cycle distribution in HT29 and LIM1215 cells transfected with NC or KD. Cells were synchronized by 48 h serum starvation, released into complete medium, and analyzed at 0, 12, and 24 h after release. The stacked bars summarize the percentages of cells in G0/G1, S, and G2/M phases for a representative experiment. Detailed representative DNA content histograms are shown in Figure S2. (F) Wound-healing assay in HT29 and LIM1215 cells transfected with NC or KD. Three straight scratches were created per well, and images were acquired at 0, 24, 48, and 72 h under serum-free conditions. The graph shows relative wound area over time, normalized to the 0-h value. Representative images at 0 and 72 h are shown. Data are presented as mean ± SD of measurements from three scratches per condition in a representative experiment. ∗*p* < 0.05, two-tailed unpaired Student’s *t* test comparing NC and KD at the corresponding time point. (G) Matrigel invasion assay in HT29 and LIM1215 cells transfected with NC or KD. Cells were seeded into three Matrigel-coated inserts per condition, and invading cells were fixed and stained with Diff-Quik at 48 h. Representative images are shown (scale bars, 200 μm). For each insert, invading cells were counted in hot-spot fields at 100× magnification, and the resulting values were normalized to the corresponding 48-h relative proliferation to generate a proliferation-adjusted invasion index. Data are presented as mean ± SD from three inserts per condition in a representative experiment. ∗*p* < 0.05, two-tailed unpaired Student’s *t* test. (H) Flow-cytometric analysis of CD133 and CD44 in HT29 cells at 48 h after transfection with NC or KD. Analysis was performed on 7-AAD-negative viable singlets under identical acquisition and gating conditions. Representative histograms and the corresponding positivity percentages from a representative experiment are shown. The flow cytometry gating strategy is provided in Figure S1.

RT-qPCR showed that *TMEM161A* mRNA levels were significantly lower in *TMEM161A* siRNA-transfected HT29 and LIM1215 cells than in the negative-control cells (*p* < 0.05, Figure 2A). Consistent with this, western blotting demonstrated a marked reduction in TMEM161A protein levels in both cell lines after siRNA transfection (Figure 2B).

These results demonstrate that *TMEM161A* expression is effectively silenced at both the mRNA and protein levels, enabling further functional assays in CRC cells.

### *TMEM161A* knockdown impairs proliferation and cell cycle progression in CRC cells

To investigate the biological function of TMEM161A in CRC cells, we performed a series of *in vitro* assays. In HT29 and LIM1215 cells, the CCK-8 assay showed that *TMEM161A* knockdown markedly decreased cell proliferation (*p* < 0.05) (Figure 2C). Consistently, colony formation assays revealed that TMEM161A-depleted cells formed significantly fewer colonies compared with control cells (*p* < 0.05) (Figure 2D). Furthermore, cell cycle analysis showed accumulation in the pre-S (G0/G1) compartment together with a reduced G2/M fraction, consistent with delayed entry into G2/M (Figure 2E; Figure S2).

### *TMEM161A* depletion is associated with reduced wound closure and invasion

In wound healing assays, *TMEM161A* knockdown delayed wound closure relative to the negative-control siRNA. The effect was significant in LIM1215 at 48–72 h (*p* < 0.05), whereas HT29 cells showed a concordant but nonsignificant trend (Figure 2F). Because the assay was performed in serum-free DMEM, the contribution of proliferation to gap closure was intended to be minimized; however, it could not be completely excluded over the 48–72 h observation period.

Using Matrigel invasion chambers, we quantified the invaded cells at 48 h and normalized the counts to the corresponding 48-h CCK-8 values to partially adjust for differences in cell growth. *TMEM161A* siRNA was associated with a reduced proliferation-adjusted invasion index in both HT29 and LIM1215 cells (mean ± SD from three inserts per condition in a representative experiment; two-tailed unpaired Student’s *t* test, *p* < 0.05) (Figure 2G). However, this correction may not fully account for the dynamic proliferation differences during the assay period.

### *TMEM161A* silencing does not enhance chemosensitivity

To assess the drug responses, *TMEM161A* siRNA-transfected and negative-control siRNA cells were treated with 5-FU, oxaliplatin, or SN-38 for 48 h. Cell viability was measured using the CCK-8 assay and is expressed relative to the corresponding untreated control (Figure S3). Across the tested dose ranges, *TMEM161A* knockdown did not produce a reproducible chemosensitizing effect in either HT29 cells or LIM1215 cells. Quantification of apparent IC_50_ values also failed to show a consistent decrease after *TMEM161A* silencing; instead, apparent IC_50_ values were increased in several conditions, including under 5-FU and oxaliplatin treatment in HT29 cells and under 5-FU treatment in LIM1215 cells (Figure S3; Table S2). Thus, under the present short-term viability assay conditions, *TMEM161A* knockdown did not show a clear chemosensitizing effect.

### *TMEM161A* knockdown reduces the CD44/CD133-positive fraction in HT29 cells

Flow cytometry at 48 h after transfection, analyzed in 7-AAD-negative viable singlets under identical acquisition and gating conditions, showed a reduced CD44/CD133-positive fraction after *TMEM161A* silencing in HT29 cells (Figure 2H; Figure S1). Using an internal negative-control threshold (>10^4^ fluorescence units for CD44), the CD44-positive fraction decreased (60.8%→48.9%), and the CD133-positive fraction likewise declined (88.6%→74.1%). Because the analysis was performed under identical acquisition and gating conditions, the comparison was focused primarily on relative differences between the NC and KD groups. Given that this analysis was limited to HT29 cells and was not complemented by sphere formation assays, these findings should be interpreted as preliminary evidence suggestive of an association with stem-like features.

### Transcriptomic analysis reveals downstream signaling changes following *TMEM161A* silencing

**Figure 3:**
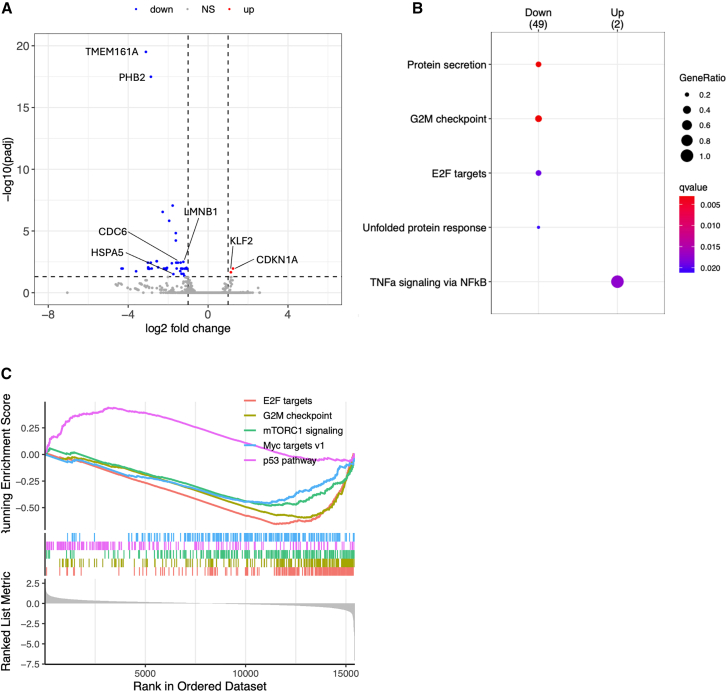
Transcriptomic changes after *TMEM161A* silencing (RNA-seq/GSEA) (A–C) RNA-seq was performed 48 h after transfection in HT29 and LIM1215 cells transfected with negative-control siRNA (NC) or TMEM161A siRNA (KD). (A) Volcano plot of differential expression (KD vs. NC) across the RNA-seq dataset. Each dot represents a gene. Vertical dashed lines indicate the cutoff of |log_2_ fold change| = 1 (fold change = 2), and the horizontal dashed line indicates the FDR (q value) cutoff of 0.05. Representative labeled genes are shown. (B) Selected Hallmark gene sets are grouped into negatively enriched (“down”) and positively enriched (“up”) categories after *TMEM161A* knockdown. Dot size indicates GeneRatio, and dot color indicates q value. (C) Representative preranked GSEA enrichment plots for the indicated MSigDB Hallmark gene sets based on the ranked differential-expression list (KD vs. NC). E2F targets, G2/M checkpoint, MYC targets v1, and mTORC1 signaling showed negative enrichment after *TMEM161A* knockdown, whereas the p53 pathway showed positive enrichment. Running enrichment score and ranked-list metric are shown. Exact NES and FDR/q values are provided in Table S1.

RNA-seq comparison of *TMEM161A*-silenced cells with negative controls revealed coordinated pathway-level shifts. Gene set enrichment analysis (GSEA) (MSigDB Hallmark; preranked log_2_ fold change) showed negative enrichment of cell cycle/growth programs, including E2F targets and G2M checkpoint (the latter denotes G2/M progression genes) and mTORC1 signaling and MYC targets v1 (e.g., E2F targets, normalized enrichment score [NES] = −2.75; G2M checkpoint, NES = −2.49; false discovery rate [FDR] q < 0.001) (Figure 3). Conversely, the p53 pathway exhibited positive enrichment (NES = 1.94; FDR q < 0.001), along with tumor necrosis factor α (TNF-α) signaling via nuclear factor kappa-light-chain-enhancer of activated B cells (NF-κB) and UV response DN (FDR q < 0.01). These transcriptomic patterns—suppression of mitotic/proliferation gene sets and enrichment of checkpoint/stress-associated responses—are consistent with the observed reduction in proliferation and delay at the G2/M phase following *TMEM161A* knockdown. Full Hallmark results and additional RNA-seq visualizations are provided in Table S1, and additional RNA-seq/GSEA displays are provided in Figure S4.

**Figure 4:**
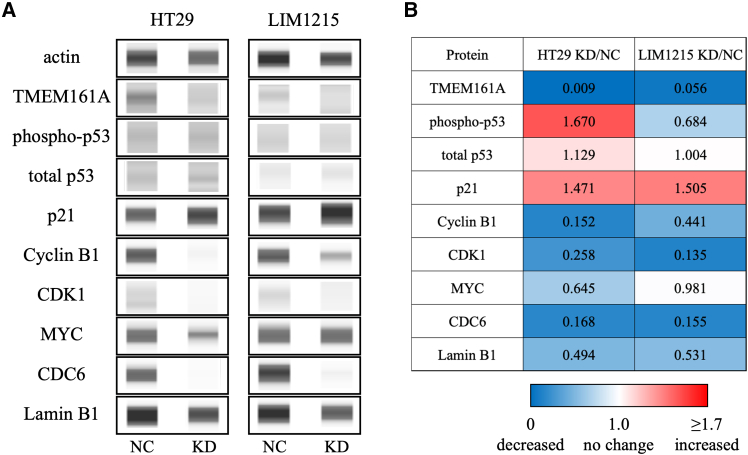
Simple Western analysis of selected downstream targets after *TMEM161A* knockdown in colorectal cancer cells (A) Simple Western images of HT29 and LIM1215 cells 48 h after transfection with negative-control siRNA (NC) or *TMEM161A* siRNA (KD). To further assess transcriptomic changes suggested by RNA-seq/GSEA analyses, selected proteins related to checkpoint/stress response and cell cycle/growth regulation, including TMEM161A, phospho-p53 (Ser15), total p53, p21, cyclin B1, CDK1, MYC, CDC6, and lamin B1, were examined. Actin is shown as a loading-related reference control. (B) Descriptive quantification of Simple Western signals using Compass-derived peak area values. Equal amounts of total protein were loaded for each sample before Simple Western analysis. Values are expressed as KD/NC ratios for each target in each cell line. Cell shading is scaled to the KD/NC ratio, with 1.0 indicating no change, blue indicating decreased signals, and red indicating increased signals relative to the corresponding NC condition, as shown in the color key. Because the Simple Western analysis was performed once for each cell line as a supportive protein-level assessment, quantification is presented without error bars or inferential statistical testing. Full-lane Simple Western overview images with molecular markers are provided in Figure S6.

To assess selected downstream targets identified by RNA-seq/GSEA at the protein level, we performed Simple Western analysis in HT29 and LIM1215 cells after *TMEM161A* knockdown. *TMEM161A* knockdown was accompanied by increased p21 expression and reduced levels of several cell cycle/growth-related proteins, including cyclin B1, CDK1, CDC6, and lamin B1, with partially variable changes in phospho-p53, total p53, and MYC between the two cell lines (Figure 4A). Descriptive quantification using Compass-derived peak area values is shown as KD/NC ratios in Figure 4B, and full-lane Simple Western overview images with molecular markers are provided in Figure S6. These findings were broadly consistent with the pathway-level changes suggested by the RNA-seq/GSEA analyses.

## Discussion

In this study, we demonstrate that TMEM161A is clinically relevant to CRC and supports malignant phenotypes *in vitro*. In a surgical cohort, high TMEM161A immunoreactivity was associated with poor survival across multiple endpoints and remained an independent prognostic factor. Similarly, siRNA-mediated silencing curtailed cell proliferation and clonogenic growth, delayed entry into the G2/M phase, and was associated with reduced Matrigel invasion and slower closure. The effect on wound closure reached significance in LIM1215 cells but not in HT29 cells. Because *TMEM161A* knockdown markedly suppressed proliferation and altered cell cycle progression, part of the reduced wound closure and invasion may be secondary to slower growth rather than a purely proliferation-independent effect. These findings are consistent with pathway-level and protein-level analyses indicating that TMEM161A supports cell cycle/growth-related programs while being associated with checkpoint and stress-related changes (Figures 3 and 4).

RNA-seq with GSEA linked TMEM161A loss to coordinated shifts: negative enrichment of cell cycle/growth programs (E2F targets, G2M checkpoint, MYC targets, and mTORC1 signaling) and positive enrichment of checkpoint/stress pathways (p53 and TNF-α/NF-κB). Protein-level analyses by Simple Western further supported this interpretation, showing reduced expression of cyclin B1, CDK1, MYC, CDC6, and lamin B1, together with increased p21 after *TMEM161A* knockdown. Phospho-p53 (Ser15) and total p53 were also evaluated; however, these findings should be interpreted cautiously, particularly in HT29 cells, because HT29 cells harbor mutant p53. Thus, our data support checkpoint/stress-associated changes rather than a definitive mechanistic conclusion of p53 pathway activation. The MYC and mTOR axes contribute to stem-like traits and therapy resistance, which is consistent with the reduction in CD44/CD133-positive fractions in HT29 cells, although this finding should be considered preliminary.

Apparent chemosensitivity to 5-FU, oxaliplatin, and SN-38 was not increased by *TMEM161A* knockdown within the tested ranges. Quantification of apparent IC_50_ values showed no reproducible chemosensitizing effect and, in some settings, showed a shift toward higher apparent IC_50_ values. A likely explanation is the altered cell cycle timing and baseline growth kinetics, because reduced proliferation can blunt short-term viability loss in assays normalized to the 0-dose condition without necessarily indicating true resistance. In this context, the observed pattern may reflect altered short-term drug response kinetics rather than genuine chemoresistance. Longer-term and cell cycle-matched assays are required to determine whether TMEM161A affects true drug sensitivity.

Mechanistically, TMEM161A is a multi-pass membrane protein with limited functional annotation in cancer. Because we did not perform interaction studies such as immunoprecipitation–mass spectrometry (IP-MS) or related assays, the link between TMEM161A and nuclear programs such as E2F/MYC should be considered speculative. Our data, therefore, support pathway-level association rather than direct molecular interaction. Together with prior reports linking AROS-29/TMEM161A to adaptive oxidative stress responses^6^ and p38 MAPK-dependent biology,^8^ our findings raise the possibility that TMEM161A may act upstream of cell cycle/growth programs through stress- or signaling-mediated mechanisms. The precise molecular intermediates remain unresolved and warrant further study.

**Figure 5:**
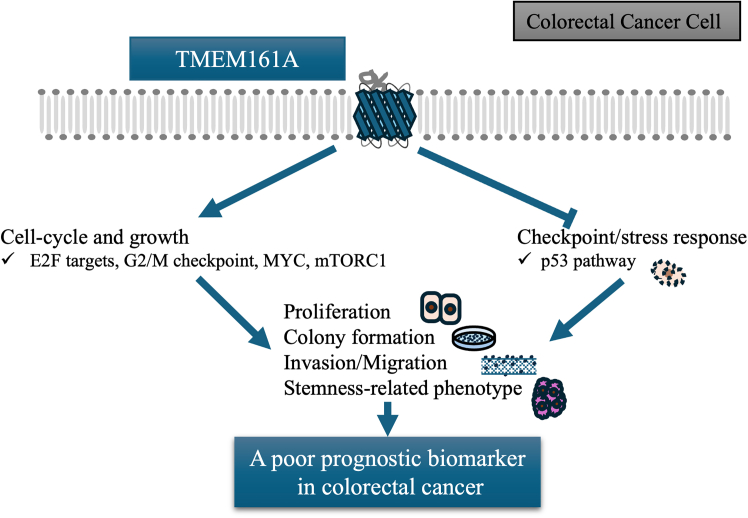
Proposed model of TMEM161A function in colorectal cancer This schematic summarizes findings consistent with a model in which elevated TMEM161A is associated with maintenance of cell cycle/growth programs including E2F targets, G2/M checkpoint, MYC targets, and mTORC1 signaling, thereby supporting proliferation, colony formation, migration/invasion-related phenotypes, and stemness-related phenotype in colorectal cancer cells. *TMEM161A* silencing was also associated with checkpoint/stress response changes, including p53-pathway enrichment at the transcriptomic level. Overall, these findings support TMEM161A as a prognostic biomarker candidate associated with a poor outcome in colorectal cancer.Symbols:→positiveassociation(inferred);⊣inverseassociation.

In summary, high TMEM161A expression in our institutional cohort was associated with poor prognosis in CRC. Functional analyses indicated that TMEM161A supports proliferation, clonogenicity, and migration/invasion-related phenotypes, while preliminary flow-cytometric findings in HT29 cells suggested its possible association with a stemness-related phenotype. At the molecular level, *TMEM161A* silencing suppressed cell cycle/growth-related programs, including E2F, G2/M checkpoint, MYC, and mTORC1 signaling, and was accompanied by checkpoint/stress-associated changes, such as increased p21 and reduced expression of several cell cycle-related proteins. In contrast, *TMEM161A* knockdown did not show a clear chemosensitizing effect under the present short-term viability assay conditions, and its impact on true drug sensitivity remains to be clarified. Taken together, these findings support TMEM161A as a biomarker candidate and a putative therapeutic node in CRC. Future studies are required to identify its upstream partners, assess its druggability, and define context-specific vulnerabilities (Figure 5).

### Limitations of the study

The limitations of this study include its single-center retrospective cohort with antibody-based scoring and functional studies of the two CRC lines. We focused primarily on knockdown experiments, and orthogonal perturbation approaches and *in vivo* validation remain to be addressed. In addition, drug response was assessed only by short-term viability assays, and apoptosis was not directly measured; therefore, the effect of *TMEM161A* knockdown on true chemosensitivity remains to be clarified. Stem-like features were assessed only by CD44/CD133 flow cytometry without complementary sphere formation assays, and, therefore, this aspect of the study should be considered preliminary. Public transcript-level analyses did not show fully consistent prognostic associations for TMEM161A across datasets and grouping strategies. Although standard TCGA-based analyses did not show a consistent adverse prognostic association, an exploratory GEPIA2 analysis of the TCGA-COAD (colon adenocarcinoma) cohort using a custom cutoff setting showed worse OS in the high-expression group (Figure S5). These findings should be interpreted cautiously because they were derived from transcript-level bulk data and were dependent on the grouping strategy. Therefore, they are considered supplementary rather than definitive external validation of our protein-based IHC findings.

## Resource availability

### Lead contact

Requests for further information, resources, and reagents should be directed to and will be fulfilled by the lead contact, Norikatsu Miyoshi (nmiyoshi@gesurg.med.osaka-u.ac.jp).

### Materials availability

This study did not generate new unique reagents or stable experimental materials. Human clinical specimens used in this study are not publicly available because of ethical and privacy restrictions. Further information about materials and resources used in this study is available from the lead contact upon reasonable request.

### Data and code availability

• RNA-seq data generated in this study are publicly available in the NCBI Gene Expression Omnibus under accession number GEO: GSE309797. Raw sequencing reads are publicly available through the Sequence Read Archive under BioProject: PRJNA1363198.^9^ These accession numbers are also listed in the key resources table.
• This paper does not report original code.
• Any additional information required to reanalyze the data reported in this paper is available from the lead contact upon reasonable request.

## Acknowledgments

We acknowledge the NGS Core Facility at the Research Institute for Microbial Diseases, University of Osaka, for the sequencing and data analysis support. This work was supported by 10.13039/501100001691JSPS KAKENHI grant number 26K11563.

## Author contributions

K.H. and N.M. conceived and designed this study; K.H., N.M., S.F., R.M., Y.T., R.H., M.T., Y.S., T.H., A.H., T.O., M.U., and H.Y. acquired the data; K.H. and N.M. analyzed and interpreted the data; K.H. and N.M. drafted the manuscript; K.H. and N.M. statistically analyzed the data; K.H., N.M., S.F., M.T., Y.S., T.H., A.H., T.O., M.U., H.Y., Y.D., and H.E. critically revised the manuscript. All the authors have read and agreed to the published version of the manuscript.

## Declaration of interests

The authors declare that they have no known competing financial interests or personal relationships that could have appeared to influence the work reported in this paper.

## Declaration of generative AI and AI-assisted technologies in the writing process

During the preparation of this work, the authors used ChatGPT (OpenAI) to improve the readability and language of the manuscript. After using this tool, the authors reviewed and edited the content as needed and take full responsibility for the content of the published article.

## STAR★Methods

### Key resources table

**Table:** 

REAGENT or RESOURCE	SOURCE	IDENTIFIER
**Antibodies**		

Rabbit polyclonal anti-TMEM161A	Proteintech	Cat#24898-1-AP
Rabbit anti-phospho-p53 (Ser15)	Cell Signaling Technology	Cat#9286 T
Rabbit anti-p53	Cell Signaling Technology	Cat#2527 T
Rabbit anti-p21	Cell Signaling Technology	Cat#2947 T
Rabbit anti-Cyclin B1	Cell Signaling Technology	Cat#12231 T
Rabbit anti-cdc2/CDK1	Cell Signaling Technology	Cat#28439 T
Rabbit anti-*c*-Myc	Cell Signaling Technology	Cat#5605 T
Rabbit anti-Cdc6	Cell Signaling Technology	Cat#3387 T
Rabbit anti-Lamin B1	Cell Signaling Technology	Cat#12586 T
Anti-actin antibody	Sigma-Aldrich	Cat#A2066
PE mouse anti-human CD44 antibody	BD Pharmingen	Cat#550989
Brilliant Violet 421 anti-human CD133 antibody	BioLegend	Cat#394012

**Biological samples**		

Human colorectal cancer FFPE tissue specimens	University of Osaka Hospital	IRB approval no. 19020-3

**Chemicals, peptides, and recombinant proteins**		

7-AAD Staining Solution	BD Pharmingen	Cat#559925

**Critical commercial assays**		

VECTASTAIN Elite ABC-HRP Kit, Peroxidase (Rabbit IgG)	Vector Laboratories	Cat#PK-6101
High-Capacity RNA-to-cDNA Kit	Applied Biosystems/Thermo Fisher Scientific	N/A
THUNDERBIRD SYBR qPCR Mix	TOYOBO	Code No. QPS-201
Bio-Rad Protein Assay Dye Reagent Concentrate	Bio-Rad Laboratories	Cat#5000006
Cell Counting Kit-8 (CCK-8)	Dojindo Laboratories	Cat#341-08001
Cell Cycle Assay Solution Blue	Dojindo Laboratories	Product code C549; Fujifilm Wako Cat#341-09601
Corning BioCoat Matrigel Invasion Chamber	Corning	Cat#354480
TruSeq Stranded mRNA Sample Preparation Kit	Illumina	N/A
Lipofectamine RNAiMAX Transfection Reagent	Invitrogen/Thermo Fisher Scientific	Cat#13778-150

**Deposited data**		

RNA-seq data generated in this study	This paper	GEO: GSE309797; BioProject: PRJNA1363198
Human reference genome GRCh38.p5	Genome Reference Consortium	GRCh38.p5
MSigDB Hallmark gene sets	MSigDB	N/A

**Experimental models: Cell lines**		

Human: HT29 colorectal cancer cell line	Laboratory stock	ATCC: HTB-38; RRID:CVCL_0320
Human: LIM1215 colorectal cancer cell line	Laboratory stock	RRID:CVCL_2574

**Oligonucleotides**		

Silencer Select Pre-Designed siRNA targeting TMEM161A	Invitrogen/Thermo Fisher Scientific	Cat#4427037; siRNA ID: s29777
Silencer Select Negative Control No. 1 siRNA	Thermo Fisher Scientific	Cat#4390843
TMEM161A qRT-PCR forward primer: GCCCAGGAGAGACAGTTCTG	This paper	N/A
TMEM161A qRT-PCR reverse primer: GCATCGAAGCCCGTGAAATC	This paper	N/A
GAPDH qRT-PCR forward primer: AGCCACATCGCTCAGACAC	This paper	N/A
GAPDH qRT-PCR reverse primer: GCCCAATACGACCAAATCC	This paper	N/A

**Software and algorithms**		

Compass for Simple Western version 6.1.0	ProteinSimple/Bio-Techne	N/A
ImageJ2 version 2.16.0/1.54p	ImageJ	https://imagej.net/software/imagej2/
Trimmomatic version 0.39	Usadel Lab	http://www.usadellab.org/cms/?page=trimmomatic
HISAT2 version 2.1.0	Kim Lab	http://daehwankimlab.github.io/hisat2/
featureCounts version 2.0.6	Subread	http://subread.sourceforge.net/
cuffdiff version 2.2.1	Cufflinks project	http://cole-trapnell-lab.github.io/cufflinks/
RNAseqChef	Etoh and Nakao^10^	https://rna-seqchef.shinyapps.io/RNAseqChef/
JMP Student Edition 18	SAS Institute Inc.	N/A

### Experimental model and study participant details

#### Human participants and clinical specimens

This retrospective study included 133 patients diagnosed with colorectal cancer who underwent primary tumor resection or amputation at the University of Osaka Hospital between January 1 and December 31, 2015. Clinical charts were reviewed from April 1, 2024, to June 30, 2025. Patients were excluded if they had undergone surgery for recurrent disease, received preoperative chemotherapy or radiotherapy, undergone transanal endoscopic microsurgery, or had incomplete pathological reports or preoperative laboratory data.

Formalin-fixed, paraffin-embedded tumor tissues obtained from surgically resected primary lesions were used for immunohistochemical analysis of TMEM161A expression. Clinicopathological variables extracted from the medical records included age, sex, tumor location, tumor size, histological grade, depth of invasion, lymph node metastasis, distant metastasis, pathological stage, serum carcinoembryonic antigen (CEA) levels, serum carbohydrate antigen 19–9 (CA19-9) levels, lymphatic invasion, venous invasion, recurrence, and survival status.

Patient age and sex were obtained from the medical records and included in the clinicopathological and survival analyses. The cohort included both male and female patients, with an overall age range of 40–86 years. Sex was evaluated as a clinicopathological variable, and its association with overall survival was assessed in Cox regression analysis. Gender identity was not available in the medical records and was therefore not analyzed in this retrospective study. Information on ancestry, race, and ethnicity was not available in the medical records and was not analyzed in this retrospective study.

After surgery, patients underwent follow-up blood examinations to assess tumor markers, including serum CEA and CA19-9, and imaging examinations, including abdominal ultrasonography, computed tomography, and chest radiography, every 3–6 months, as recommended in the Japanese Society for Cancer of the Colon and Rectum guidelines. Patients with stage III or IV disease who underwent R0 resection received adjuvant postoperative chemotherapy after providing informed consent, in accordance with the guidelines.

This study was conducted in accordance with the Declaration of Helsinki and was approved by the Institutional Review Board of the University of Osaka Hospital (approval no. 19020–3; approved on May 31, 2019). Written informed consent was obtained from all participants prior to inclusion in the study.

#### Cell lines and culture conditions

Human colorectal cancer cell lines HT29 and LIM1215 were used for *in vitro* experiments. HT29 and LIM1215 were selected from the colorectal cancer cell lines available in our laboratory because they showed relatively high endogenous TMEM161A expression and efficient siRNA-mediated knockdown in preliminary experiments. HT29 is microsatellite stable and harbors BRAF V600E and TP53 R273H with wild-type KRAS, whereas LIM1215 is microsatellite unstable and is wild type for KRAS, BRAF, and TP53.

HT29 and LIM1215 cells were maintained in Dulbecco’s modified Eagle’s medium (DMEM; Thermo Fisher Scientific Inc., Waltham, MA, USA) supplemented with 10% fetal bovine serum (FBS; Thermo Fisher Scientific Inc.). Cells were cultured at 37°C in a humidified atmosphere containing 5% CO2 in air. HT29 and LIM1215 cells were obtained from laboratory stocks that had been maintained in our laboratory and were not independently authenticated for this study. Formal mycoplasma testing was not performed for this study.

### Method details

#### Immunohistochemistry

Immunohistochemistry was performed on formalin-fixed, paraffin-embedded tissues obtained from surgically resected primary colorectal cancer lesions. Tissue sections were incubated overnight at 4°C with an anti-TMEM161A rabbit polyclonal antibody (cat. no. 24898-1-AP, Proteintech, Rosemont, IL, USA) at a dilution of 1:200. After incubation with the primary antibody, the sections were treated for 30 min with a biotinylated secondary antibody (Vectastain Universal Elite; Vector Laboratories, Burlingame, CA, USA). Color development was achieved using ImmPACT DAB substrate (Vector Laboratories) for 3 min, and all slides were counterstained with hematoxylin. Normal brain tissue served as a positive control for TMEM161A, in accordance with the instructions provided in the antibody package insert.

TMEM161A staining intensity was evaluated based on cytoplasmic staining in hotspot areas of the tumor tissue. Staining intensity was classified into four categories: 0, no staining; 1+, weak staining; 2+, moderate staining; and 3+, strong staining. Based on these scores, cases were categorized as TMEM161A-low expression (score 0 or 1+) or TMEM161A-high expression (score 2+ or 3+). The dichotomous cutoff was chosen primarily based on pathological interpretability, distinguishing absent/weak staining from moderate/strong staining. For the main scoring of the full cohort, the evaluators did not refer to clinical outcomes at the time of evaluation. The scoring was reviewed in a prognosis-blinded manner by three coauthors. Formal interobserver agreement statistics were not calculated in this study.

#### siRNA-mediated knockdown

HT29 and LIM1215 cells were used for siRNA-mediated knockdown of TMEM161A. Duplex RNAs targeting human TMEM161A (Silencer Select TMEM161A siRNA, ID s29777) and a nontargeting negative-control siRNA (Silencer Select Negative Control No. 1; both from Thermo Fisher Scientific, Waltham, MA, USA) were used. Cells were transfected with siRNA at a final concentration of 83 nM using Lipofectamine RNAiMAX (Thermo Fisher Scientific) in glucose-free Opti-MEM (Thermo Fisher Scientific), according to the manufacturer’s protocol. Knockdown efficiency was evaluated by quantitative reverse transcription-PCR and Simple Western analysis.

#### Quantitative reverse transcription-PCR

Total RNA was isolated from cultured cells using TRIzol reagent (Thermo Fisher Scientific), according to the manufacturer’s protocol. First-strand cDNA was generated from 1 μg of total RNA using a High-Capacity RNA-to-cDNA Kit (Applied Biosystems; Thermo Fisher Scientific), according to the manufacturer’s protocol. Quantitative PCR was performed using THUNDERBIRD SYBR qPCR Mix (TOYOBO, Osaka, Japan) with ROX reference dye on a QuantStudio 7 Flex Real-Time PCR System (Applied Biosystems; Thermo Fisher Scientific).

The primer sequences were as follows:

TMEM161A forward: 5′-GCCCAGGAGAGACAGTTCTG-3′

TMEM161A reverse: 5′-GCATCGAAGCCCGTGAAATC-3′

GAPDH forward: 5′-AGCCACATCGCTCAGACAC-3′

GAPDH reverse: 5′-GCCCAATACGACCAAATCC-3′

Relative expression levels were calculated using the 2^−ΔΔCt^ method, with GAPDH used as the endogenous control.

#### Simple Western analysis

Protein expression was analyzed using a capillary-based automated immunoassay system (Simple Western; Jess, ProteinSimple, San Jose, CA, USA), according to the manufacturer’s instructions. Cell lysates were prepared in radioimmunoprecipitation assay buffer containing protease and phosphatase inhibitors, and protein concentrations were determined using a Bio-Rad Bradford protein assay with BSA standards. Absorbance was measured at 595 nm. Samples were denatured and loaded into the Jess system.

The following primary antibodies were used: TMEM161A (Proteintech; 1:20), phospho-p53 (Ser15) (Cell Signaling Technology, #9286 T; 1:10), p53 (Cell Signaling Technology, #2527 T; 1:5), p21 (Cell Signaling Technology, #2947 T; 1:25), Cyclin B1 (Cell Signaling Technology, #12231 T; 1:10), cdc2/CDK1 (Cell Signaling Technology, #28439 T; 1:5), c-Myc (Cell Signaling Technology, #5605 T; 1:100), Cdc6 (Cell Signaling Technology, #3387 T; 1:10), Lamin B1 (Cell Signaling Technology, #12586 T; 1:25), and actin (Sigma-Aldrich, A2066; 1:50 for TMEM161A validation and 1:100 for downstream protein analyses). HRP-conjugated secondary antibodies supplied by ProteinSimple were used for detection.

Signals were analyzed using Compass for Simple Western software. Equal amounts of total protein were loaded for each sample. Compass-derived peak area values were used for descriptive quantification and expressed relative to the corresponding negative-control condition for each target. The Simple Western analysis was performed once for each cell line as a supportive protein-level assessment.

#### Cell proliferation assay

HT29 and LIM1215 cells transfected with either TMEM161A-targeting siRNA or negative-control siRNA were plated at 1.0 × 10ˆ5 cells per well in 6-well plates. After 24 h, cells were trypsinized and reseeded into 96-well plates at 1,000 cells per well. Cell proliferation was evaluated using a Cell Counting Kit-8 assay (CCK-8; Dojindo Molecular Technologies, Inc., Kumamoto, Japan) at 0, 24, 48, and 72 h after reseeding. Following incubation with 10% CCK-8 solution at 37°C for 2 h, absorbance at 450 nm was measured using a microplate reader (Bio-Rad Laboratories, Hercules, CA, USA). Proliferation was quantified as the fold change in absorbance relative to the 0-h time point.

#### Cell cycle assay

Cell cycle analysis was performed using Cell Cycle Assay Solution Blue (Dojindo Molecular Technologies). HT29 and LIM1215 cells transfected with TMEM161A siRNA or negative-control siRNA were synchronized by serum starvation in DMEM without FBS for 48 h. Cells were then released into complete medium and harvested at 0, 12, and 24 h after release. After staining with the assay solution according to the manufacturer’s protocol, DNA content was analyzed using a BD FACSCanto II flow cytometer (BD Biosciences, San Jose, CA, USA). The proportions of cells in the G0/G1, S, and G2/M phases were calculated.

#### Colony formation assay

HT29 and LIM1215 cells transfected with TMEM161A siRNA or negative-control siRNA were seeded in 6-well plates at 100 cells per well and cultured under standard conditions. On day 10, colonies containing 50 or more cells were fixed with methanol and stained with 0.5% crystal violet. Colonies were counted under a stereomicroscope.

#### Wound healing assay

For wound healing assays, 1 × 10ˆ5 cells were seeded into 6-well plates and cultured in complete medium until the monolayer reached approximately 90% confluence. The medium was then replaced with serum-free DMEM, and three straight scratches were drawn across each well using a 200-μL pipette tip. Detached cells were removed by two gentle washes with phosphate-buffered saline, after which fresh serum-free medium was added. Marks were made on the underside of the plate near the scratched region to record the same field over time. Images of the wounds were acquired at 0, 24, 48, and 72 h using an inverted microscope.

For image analysis, no dedicated wound-healing plugin was used. Wound areas were manually quantified in Fiji/ImageJ (National Institutes of Health, Bethesda, MD, USA) using threshold-based segmentation followed by the Analyze Particles function. The residual wound area at each time point was measured as area (μmˆ2), and the relative wound area was calculated as Area_t/Area_0 h × 100. The same analysis parameters were applied across images within each experiment.

#### Matrigel invasion assay

HT29 and LIM1215 cells transfected with TMEM161A siRNA or negative-control siRNA were suspended in serum-free DMEM and seeded at 2.5 × 10ˆ4 cells per insert into 24-well Matrigel-coated Transwell inserts (Corning BioCoat Matrigel Invasion Chamber, cat. no. 354480). DMEM containing 20% FBS was added to the lower chamber as a chemoattractant. After 48 h at 37°C in 5% CO2, non-invading cells were removed from the upper membrane with a cotton swab. Invading cells on the lower surface were fixed and stained with Diff-Quik (Sysmex Corp., Kobe, Japan), according to the manufacturer’s instructions. Invading cells were counted in three nonoverlapping hotspot fields at ×100 magnification, and the mean count per insert was used for analysis.

To partially adjust for the effect of differential growth, invasion counts were normalized to the corresponding 48-h relative proliferation measured in parallel cultures and reported as a proliferation-adjusted invasion index.

#### Chemosensitivity assay

Chemosensitivity was evaluated after siRNA transfection. CRC cells were passaged on day 0 and transfected on day 1. On day 2, cells were reseeded into 96-well plates at 2.0 × 10ˆ3 cells per 100 μL per well. On day 3, 5-fluorouracil, oxaliplatin, or SN-38 was added in a serial dilution of 0, ×1/256, ×1/64, ×1/16, ×1/4, and ×1. The ×1 concentrations were 8,000 μM for 5-fluorouracil, 800 μM for oxaliplatin, and 80 μM for SN-38.

After 48 h of drug exposure, cell viability was measured using CCK-8 solution at 10% v/v for 2 h at 37°C, and absorbance at 450 nm was recorded. For each transfection condition, viability at each dose was normalized to the 0-dose value on the same plate and expressed as relative viability. Apparent IC50 values were estimated from short-term viability data by separately fitting a dose-response curve for each well.

#### Flow cytometric analysis

To assess cancer stem-like fractions, cells were stained for surface markers and analyzed by flow cytometry. Cells were detached with trypsin without ethylenediaminetetraacetic acid, washed twice in phosphate-buffered saline, and incubated with the following fluorophore-conjugated antibodies and viability dye: PE-labeled mouse anti-human CD44 (BD Pharmingen, BD Biosciences), Brilliant Violet 421-labeled anti-human CD133 (BioLegend, San Diego, CA, USA), and 7-aminoactinomycin D (7-AAD; BD Pharmingen, BD Biosciences). After staining, samples were run on a BD FACSCanto II flow cytometer (BD Biosciences).

Cells were analyzed under identical acquisition and gating conditions. The gating strategy is provided in Figure S1. Comparisons were intended primarily for relative comparison between negative-control and TMEM161A-knockdown cells rather than emphasis on absolute positivity values.

#### RNA sequencing and transcriptomic analysis

RNA sequencing was performed in HT29 and LIM1215 cells transfected with TMEM161A-targeting siRNA or negative-control siRNA. Library preparation was performed using the TruSeq Stranded mRNA Sample Preparation Kit (Illumina, San Diego, CA, USA), according to the manufacturer’s instructions. Sequencing was performed on a DNBSEQ-G400 sequencer (MGI Tech Co., Ltd., Shenzhen, China) in 100-base single-read mode.

After adapter sequence removal using Trimmomatic version 0.39, reads were aligned to the human reference genome GRCh38.p5 using HISAT2 version 2.1.0. Gene-level expression was quantified using featureCounts version 2.0.6, and differential expression analysis was performed using cuffdiff version 2.2.1.

For downstream transcriptomic exploration and visualization of processed RNA-seq data, RNAseqChef was used.^10^ Selected displays, including PCA/MDS plots, MA plots, heatmaps, and enrichment-network style summaries, were generated or organized using RNAseqChef. Gene set enrichment analysis was performed using the MSigDB Hallmark gene sets based on the ranked differential-expression list comparing TMEM161A-knockdown cells with negative-control cells. Full RNA-seq data availability is described in the Data and code availability subsection of resource availability.

### Quantification and statistical analysis

Statistical analyses were performed using Microsoft Excel and JMP Student Edition 18 (SAS Institute Inc., Cary, NC, USA). Continuous variables are presented as medians with ranges or as means ± standard deviations (SDs), as appropriate. Categorical variables were compared using the chi-square test or Fisher’s exact test, as appropriate.

Survival curves were estimated using the Kaplan–Meier method and compared using the log rank test. Overall survival, disease-free survival, cancer-specific survival, and recurrence-free survival were analyzed according to TMEM161A expression status. Cox proportional hazards regression analysis was performed to evaluate prognostic factors for overall survival. Variables with clinical relevance and/or statistical significance in univariate analysis were included in multivariate Cox regression analysis. Hazard ratios (HRs), 95% confidence intervals (CIs), and *p* values are reported.

For *in vitro* experiments comparing two groups, statistical comparisons were performed using a two-tailed unpaired Student’s *t* test unless otherwise specified. For qRT-PCR, relative expression levels were calculated using the 2^−ΔΔCt^ method after normalization to GAPDH. For cell proliferation assays, absorbance values were normalized to the 0-h time point and expressed as fold changes. For wound-healing assays, wound areas were quantified using Fiji/ImageJ and expressed as relative wound area, calculated as Area_t/Area_0 h × 100. For Matrigel invasion assays, invading cell counts were normalized to the corresponding 48-h relative proliferation measured in parallel cultures and expressed as a proliferation-adjusted invasion index.

For chemosensitivity assays, cell viability at each drug concentration was normalized to the 0-dose value within the same transfection condition. Apparent IC50 values were estimated from short-term viability data by separately fitting dose-response curves for each well. Comparisons of apparent IC50 values between negative-control and TMEM161A-knockdown cells were performed using log10-transformed apparent IC50 values.

For Simple Western analysis, Compass-derived peak area values were used for descriptive quantification. Values were expressed relative to the corresponding negative-control condition for each target. Because the Simple Western analysis was performed once for each cell line as a supportive protein-level assessment, quantification was presented descriptively without error bars or inferential statistical testing.

For RNA-seq analysis, differential expression analysis was performed using cuffdiff. Gene set enrichment analysis was performed using the MSigDB Hallmark gene sets based on the ranked differential-expression list comparing TMEM161A-knockdown cells with negative-control cells. Normalized enrichment scores (NESs), adjusted *p* values, and false discovery rate (FDR) q values were reported. Gene sets with FDR q values below the indicated thresholds were considered enriched.

All statistical tests were two-sided unless otherwise stated. A *p* value <0.05 was considered statistically significant. Details of the number of samples, wells, inserts, scratches, or technical replicates used for each experiment are provided in the corresponding figure legends.

### Additional resources

This study is not associated with a clinical trial. No additional resources, such as a study website, external protocol, or registered clinical resource, were generated as part of this study.
