# Supplementary material for: High TMEM161A expression drives malignant phenotypes and predicts poor prognosis in colorectal cancer
Source: iScience. 2026 Jun 27;29(7):116550. doi: 10.1016/j.isci.2026.116550 (PMC13378339; doi:10.1016/j.isci.2026.116550)
Supplement: Document S1. Figures S1–S6 and Tables S1 and S2 [file mmc1.pdf]

iScience, Volume *29*

Supplemental information

High TMEM161A expression drives malignant phenotypes and predicts poor prognosis in colorectal cancer

Kengo Haruna, Norikatsu Miyoshi, Shiki Fujino, Rie Mizumoto, Yuki Toyoda, Rie Hayashi, Mitsunobu Takeda, Yuki Sekido, Tsuyoshi Hata, Atsushi Hamabe, Takayuki Ogino, Mamoru Uemura, Hirofumi Yamamoto, Hidetoshi Eguchi, and Yuichiro Doki

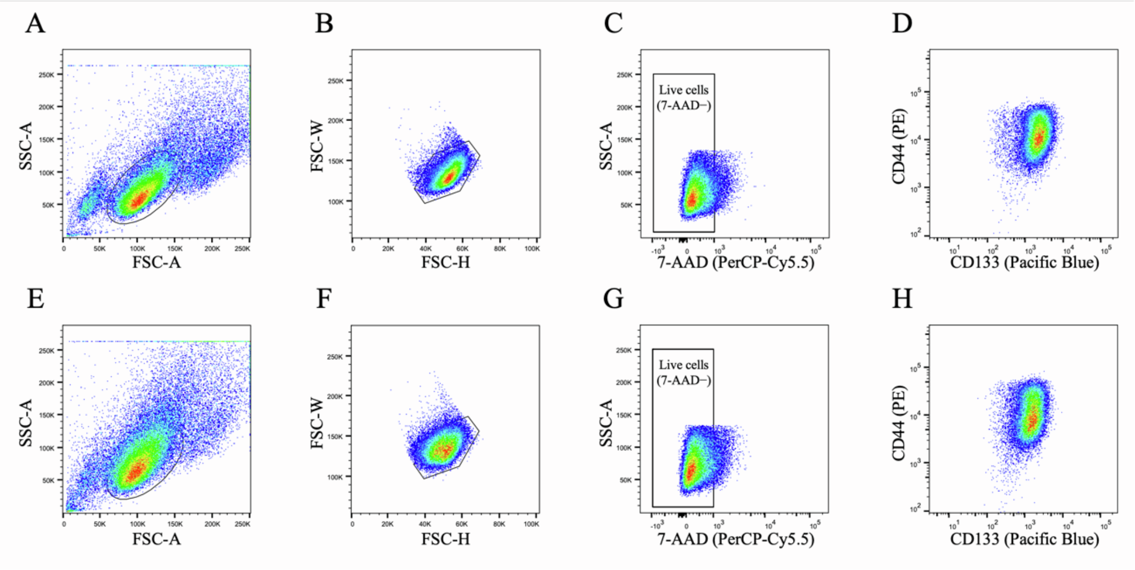

Supplementary Figure S1. Flow cytometry gating strategy for CD44/CD133 analysis in HT29 cells.

Representative flow cytometry gating strategy used for analysis of CD44/CD133-positive fractions in HT29 cells transfected with negative-control siRNA (NC) or TMEM161A siRNA (KD). Panels A–D show the gating sequence for the NC sample, and panels E–H show the corresponding gating sequence for the KD sample. Cells were sequentially gated by forward/side scatter to exclude debris (A, E), singlet gating (B, F), and selection of 7-AAD-negative viable cells (C, G), followed by analysis of CD44 and CD133 expression in the viable singlet population (D, H). Identical acquisition, gating, and positivity-threshold conditions were applied to the NC and KD samples. These plots are shown to document the gating strategy and to support relative comparison between NC and KD rather than emphasis on absolute positivity values.

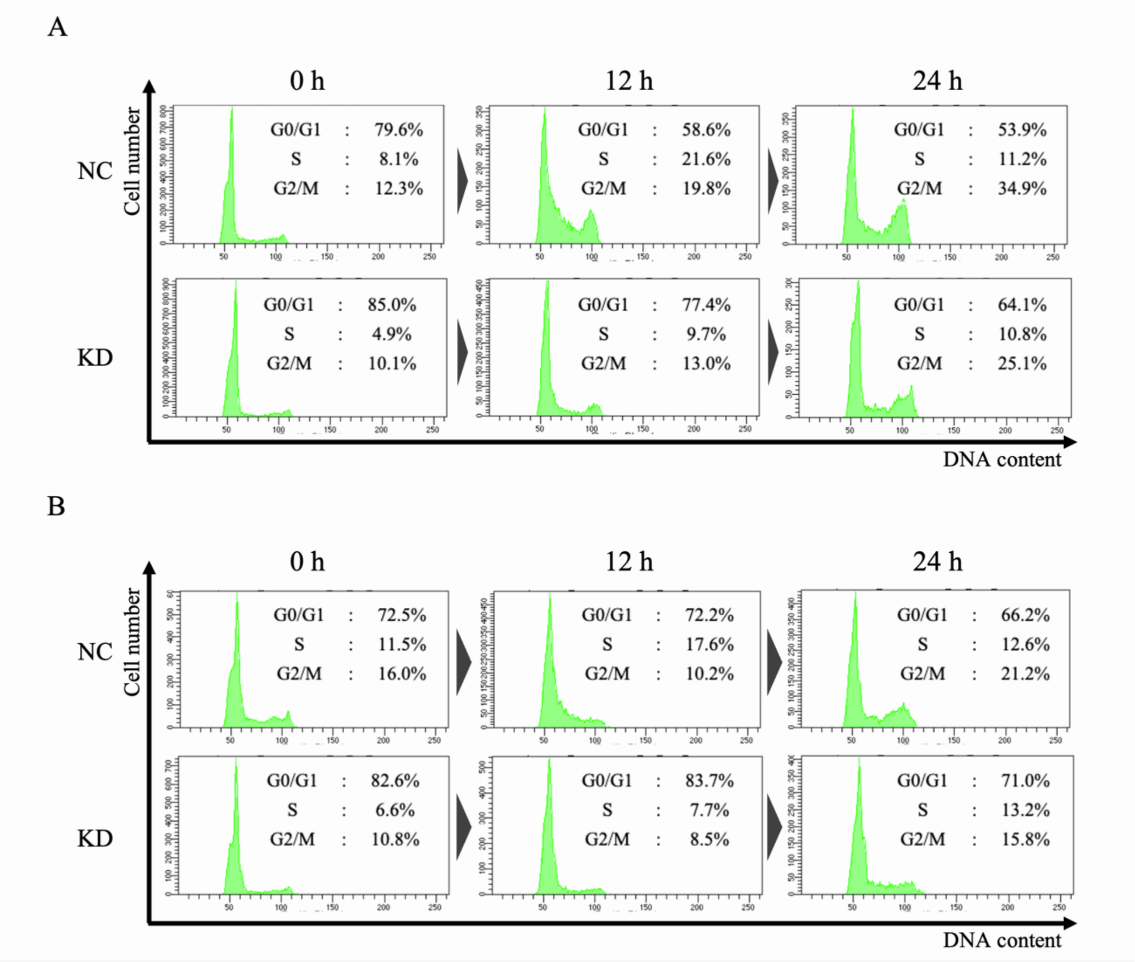

Supplementary Figure S2. Flow-cytometric analysis of cell-cycle distribution after TMEM161A knockdown.

(A) HT29 and (B) LIM1215. Cells were transfected with NC or TMEM161A siRNA (KD), serum-starved for 48 h to synchronize, released into complete medium, and collected at 0, 12, and 24 h. DNA content was stained with Cell Cycle Assay Solution Blue, and singlet cells were analyzed by flow cytometry. Representative DNA-count histograms show the percentages of cells in G0/G1, S, and G2/M at each time point (values indicated on plots). These representative histograms are consistent with a shift toward a higher G0/G1 fraction and reduced G2/M progression after TMEM161A silencing at 12–24 h in both cell lines. These histograms correspond to the summary shown in Figure 2E.

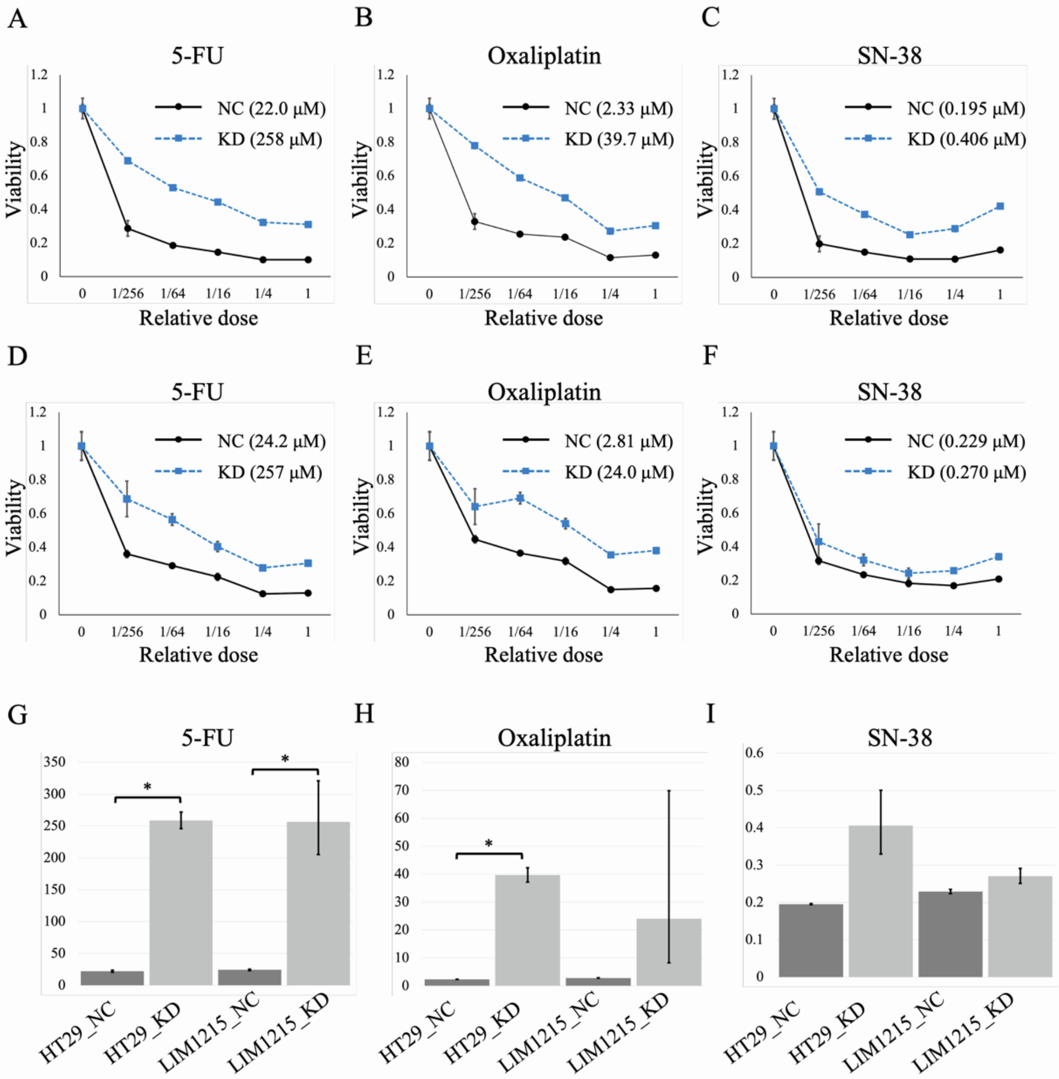
A
5-FU
NC (22.0 μM)
KD (258 μM)
Viability
Relative dose
B
Oxaliplatin
NC (2.33 μM)
KD (39.7 μM)
C
SN-38
NC (0.195 μM)
KD (0.406 μM)
D
5-FU
NC (24.2 μM)
KD (257 μM)
E
Oxaliplatin
NC (2.81 μM)
KD (24.0 μM)
F
SN-38
NC (0.229 μM)
KD (0.270 μM)
0
1/256
1/64
1/16
1/4
1
G
5-FU
H
Oxaliplatin
I
SN-38
HT29_NC
HT29_KD
LIM1215_NC
LIM1215_KD

Supplementary Figure S3. Chemosensitivity after TMEM161A knockdown.

(A–C) HT29 and (D–F) LIM1215 cells transfected with negative-control siRNA (NC) or TMEM161A siRNA (KD) were reseeded at 2.0 × 10^3 cells/well and exposed for 48 h to the indicated relative doses of 5-FU (A, D), oxaliplatin (B, E), and SN-38 (C, F) (×1 = 5-FU, 8,000 μM; oxaliplatin, 800 μM; SN-38, 80 μM). Viability was measured by CCK-8 and normalized to the 0-dose value within each transfection group. Apparent IC50 values are indicated in parentheses. Data are presented as mean ± SD from three wells per dose in a representative experiment.

(G–I) Apparent IC50 summaries for 5-FU (G), oxaliplatin (H), and SN-38 (I) in HT29 and LIM1215 cells, derived from the same dose-response experiments shown in panels A–F. Bars represent geometric mean apparent IC50 values estimated by separately fitting dose-response curves for each well (three wells per condition in a representative experiment). Error bars indicate SEM. P values were calculated from log10-transformed apparent IC50 values using a two-tailed unpaired Student's t-test. Because TMEM161A knockdown markedly suppressed baseline proliferation, these apparent IC50 estimates should be interpreted cautiously and do not necessarily indicate true chemoresistance.

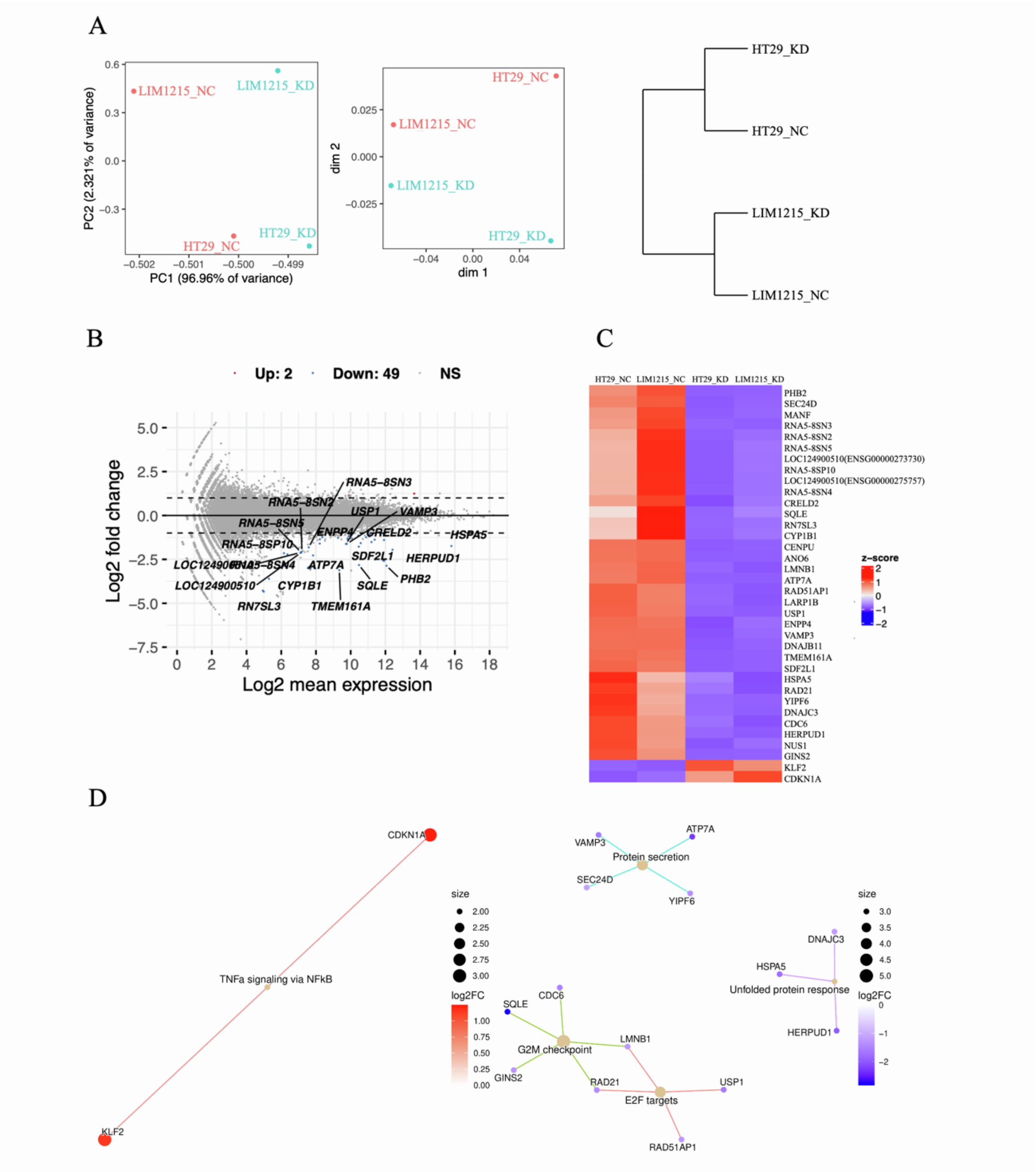
A
PC2 (2.321% of variance)
PC1 (96.96% of variance)
LIM1215_NC
LIM1215_KD
HT29_NC
HT29_KD
dim 1
dim 2
B
Up: 2
Down: 49
NS
Log2 fold change
Log2 mean expression
RNA5-8SN3
RNA5-8SN2
RNA5-8SN5
RNA5-8SP10
RNA5-8SN4
USP1
VAMP3
ENPP4
CRELD2
HSPA5
SDF2L1
HERPUD1
ATP7A
PHB2
SQLE
CYP1B1
LOC124900510
RN7SL3
TMEM161A
C
z-score
PHB2
SEC24D
MANF
RNA5-8SN3
RNA5-8SN2
RNA5-8SN5
LOC124900510(ENSG00000273730)
RNA5-8SP10
LOC124900510(ENSG00000275757)
RNA5-8SN4
CRELD2
SQLE
RN7SL3
CYP1B1
CENPU
ANO6
LMNB1
ATP7A
RAD51AP1
LARP1B
USP1
ENPP4
VAMP3
DNAJB11
TMEM161A
SDF2L1
HSPA5
RAD21
YIPF6
DNAJC3
CDC6
HERPUD1
NUS1
GINS2
KLF2
CDKN1A
D
CDKN1A
TNFa signaling via NFkB
KLF2
VAMP3
ATP7A
Protein secretion
SEC24D
YIPF6
size
log2FC
SQLE
CDC6
G2M checkpoint
LMNB1
GINS2
RAD21
E2F targets
USP1
RAD51AP1
DNAJC3
HSPA5
Unfolded protein response
HERPUD1

Supplementary Figure S4. Quality control and extended RNA-seq/GSEA displays.

(A) Principal component analysis (PCA, left), multidimensional scaling (MDS, middle), and hierarchical clustering (right) of the four RNA-seq samples (HT29_NC, HT29_KD, LIM1215_NC, and LIM1215_KD). NC, negative-control siRNA; KD, TMEM161A siRNA.

(B) MA plot of differentially expressed genes with representative gene labels. Genes meeting the predefined differential expression criteria are highlighted, and the numbers of upregulated and downregulated genes are indicated.

(C) Heatmap of representative differentially expressed genes across the four samples, shown as row-wise z-scores.

(D) Enrichment network / leading-edge display linking representative Hallmark gene sets and associated genes.

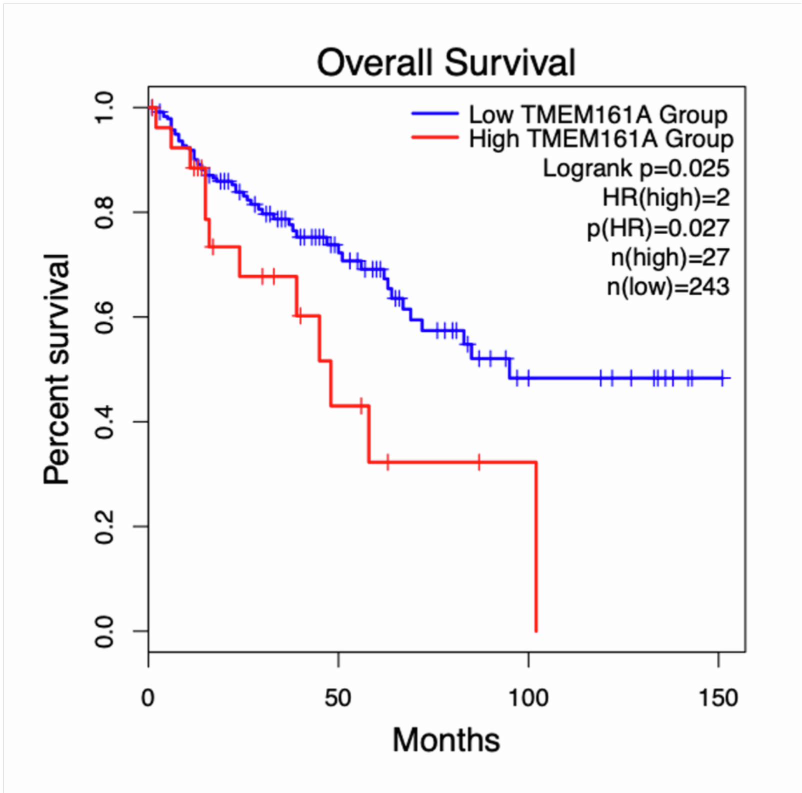

Supplementary Figure S5. Exploratory GEPIA2 survival analysis of TMEM161A expression in the COAD cohort.

Kaplan–Meier analysis of overall survival in the GEPIA2 COAD cohort using a custom cutoff. Patients were dichotomized with the high-expression group defined as the top 10% of cases and the low-expression group as the remaining 90%. Hazard ratio, log-rank p value, and the number of patients in each group are shown on the plot.

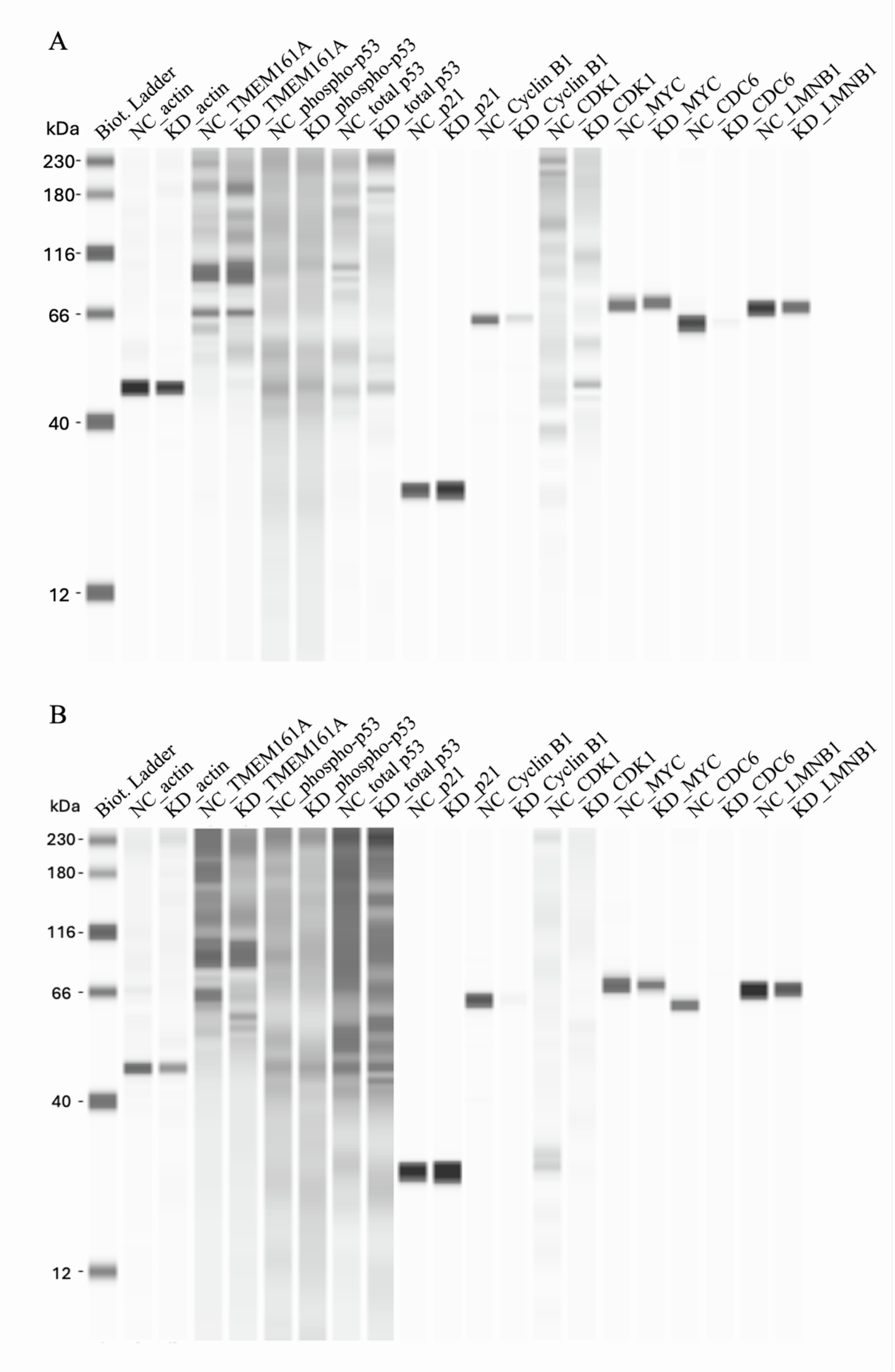
A
kDa
230
180
116
66
40
12
Biot. Ladder
NC_actin
KD_actin
NC_TMEM161A
KD_TMEM161A
NC_phospho-p53
KD_phospho-p53
NC_total p53
KD_total p53
NC_p21
KD_p21
NC_Cyclin B1
KD_Cyclin B1
NC_CDK1
KD_CDK1
NC_MYC
KD_MYC
NC_CDC6
KD_CDC6
NC_LMNB1
KD_LMNB1
B
kDa
230
180
116
66
40
12
Biot. Ladder
NC_actin
KD_actin
NC_TMEM161A
KD_TMEM161A
NC_phospho-p53
KD_phospho-p53
NC_total p53
KD_total p53
NC_p21
KD_p21
NC_Cyclin B1
KD_Cyclin B1
NC_CDK1
KD_CDK1
NC_MYC
KD_MYC
NC_CDC6
KD_CDC6
NC_LMNB1
KD_LMNB1

Supplementary Figure S6. Full-lane Simple Western overview images with molecular markers.

Full lane-view images from the Simple Western analysis corresponding to Figure 4 are shown for HT29 (A) and LIM1215 (B) cells 48 h after transfection with negative-control siRNA (NC) or TMEM161A siRNA (KD). Molecular weight markers are shown on the left of each panel. The indicated proteins include actin, TMEM161A, phospho-p53 (Ser15), total p53, p21, Cyclin B1, CDK1, MYC, CDC6, and Lamin B1. For each target, the NC and KD lanes were displayed using the same brightness and contrast settings, whereas display settings were adjusted separately between different targets to improve visualization. Compass-derived peak area values were used for quantification in Figure 4B. When multiple signals were visible, the quantified signal was selected based on the Compass peak assignment and the antibody-specific apparent molecular weight. The quantified apparent molecular weights were approximately 48 kDa for actin, 65–69 kDa for TMEM161A, 47–49 kDa for phospho-p53 (Ser15) and total p53, 29 kDa for p21, 64–65 kDa for Cyclin B1, 30–39 kDa for CDK1, 72–75 kDa for MYC, 63–64 kDa for CDC6, and 68–71 kDa for Lamin B1. Signal intensity comparisons should therefore be interpreted within each NC/KD pair for the same target. The Simple Western analysis was performed once for each cell line as a supportive protein-level assessment. NC, negative control; KD, TMEM161A knockdown.

Supplementary Table S1. Hallmark GSEA results after TMEM161A silencing.

Gene_set_name	NES	p.adjust	q value
E2F targets	–2.751226	0.0000000	0.0000000
G2M checkpoint	–2.492304	0.0000000	0.0000000
mTORC1 signaling	–2.017487	0.0000009	0.0000007
p53 pathway	1.936332	0.0000009	0.0000007
Myc targets v1	–1.918793	0.0000115	0.0000082
Unfolded protein response	–1.979998	0.0000344	0.0000244
Mitotic spindle	–1.630636	0.0027412	0.0019460
UV response dn	1.595163	0.0050531	0.0035873
Protein secretion	–1.698907	0.0057287	0.0040669
TNFα signaling via NF-κB	1.463679	0.0096595	0.0068574
Spermatogenesis	–1.640644	0.0132448	0.0094027
TGF beta signaling	1.557100	0.0414195	0.0294043

RNA-seq of HT29 and LIM1215 cells was performed 48 h after transfection with TMEM161A or negative-control siRNA. Genes ranked by differential expression (siRNA vs. NC) were analyzed against the MSigDB Hallmark collection. The table reports the NES, p. adjusted (BH-adjusted p), and q values (FDR). Negative NES indicates depletion in TMEM161A-silenced cells, whereas positive NES indicates enrichment. Additional parameters are described in the Methods section.

Supplementary Table S2. Apparent IC50 estimates and 95% confidence intervals after TMEM161A knockdown.

**Cell line**	**Drug**	**NC apparent IC50 (95% CI), μM**	**KD apparent IC50 (95% CI), μM**	**p value (log10IC50)**
HT29	5-FU	22.0 (16.5–29.3)	259 (208–322)	< 0.001
HT29	Oxaliplatin	2.33 (2.25–2.41)	39.7 (30.1–52.3)	< 0.001
HT29	SN-38	0.195 (0.189–0.202)	0.406 (0.165–0.997)	0.072
LIM1215	5-FU	24.2 (19.8–29.6)	256 (97.8–672)	0.007
LIM1215	Oxaliplatin	2.81 (2.57–3.08)	24.0 (0.244–2370)	0.182
LIM1215	SN-38	0.229 (0.205–0.255)	0.270 (0.196–0.372)	0.146

Apparent IC50 values were estimated from short-term CCK-8 viability assays in HT29 and LIM1215 cells transfected with negative control siRNA (NC) or TMEM161A siRNA (KD). Values are shown as geometric mean apparent IC50 with 95% confidence intervals. P values were calculated from log10-transformed apparent IC50 values for NC vs KD comparisons within each cell line and drug condition. Because TMEM161A knockdown markedly suppressed baseline proliferation, these apparent IC50 estimates should be interpreted cautiously and do not necessarily indicate true chemoresistance.
